# A Phase 0 Study to Assess the Biodistribution and Pharmacokinetics of a Radiolabeled Antibody Targeting Human Kallikrein 2 in Participants with Metastatic Castration-Resistant Prostate Cancer

**DOI:** 10.2967/jnumed.124.267416

**Published:** 2024-07

**Authors:** Neeta Pandit-Taskar, Joseph A. O’Donoghue, Dushen Chetty, Steven Max, Danielle Wanik, Ohad Ilovich, Michael Russell, Tenzin Nyima, Chaitanya R. Divgi, Margaret Yu, Michael J. Morris

**Affiliations:** 1 Department of Radiology, Memorial Sloan Kettering Cancer Center, New York, New York;; 2 Department of Radiology, Weill Cornell Medical Center, New York, New York;; 3 Department of Medical Physics, Memorial Sloan Kettering Cancer Center, New York, New York;; 4 Janssen Research & Development, LLC, Spring House, Pennsylvania;; 5 Invicro, LLC, Boston, Massachusetts;; 6 Memorial Sloan Kettering Cancer Center, New York, New York;; 7 Genitourinary Oncology Service, Memorial Sloan Kettering Cancer Center, New York, New York; and; 8 Department of Medicine, Weill Cornell Medicine, New York, New York

**Keywords:** mCRPC, hK2, KLK2, h11B6

## Abstract

Despite the inclusion of multiple agents within the prostate cancer treatment landscape, new treatment options are needed to address the unmet need for patients with metastatic castration-resistant prostate cancer (mCRPC). Although prostate-specific membrane antigen is the only cell-surface target to yield clinical benefit in men with advanced prostate cancer, additional targets may further advance targeted immune, cytotoxic, radiopharmaceutical, and other tumor-directed therapies for these patients. Human kallikrein 2 (hK2) is a novel prostate-specific target with little to no expression in nonprostate tissues. This first-in-human phase 0 trial uses an ^111^In-radiolabeled anti-hK2 monoclonal antibody, [^111^In]-DOTA-h11B6, to credential hK2 as a potential target for prostate cancer treatment. **Methods:** Participants with progressive mCRPC received a single infusion of 2 mg of [^111^In]-DOTA-h11B6 (185 MBq of ^111^In), with or without 8 mg of unlabeled h11B6 to assess antibody mass effects. Sequential imaging and serial blood samples were collected to determine [^111^In]-DOTA-h11B6 biodistribution, dosimetry, serum radioactivity, and pharmacokinetics. Safety was assessed within a 2-wk follow-up period from the time of [^111^In]-DOTA-h11B6 administration. **Results:** Twenty-two participants received [^111^In]-DOTA-h11B6 and are included in this analysis. Within 6–8 d of administration, [^111^In]-DOTA-h11B6 visibly accumulated in known mCRPC lesions, with limited uptake in other organs. Two treatment-emergent adverse events unrelated to treatment occurred, including tumor-related bleeding in 1 patient, which led to early study discontinuation. Serum clearance, biodistribution, and tumor targeting were independent of total antibody mass (2 or 10 mg). **Conclusion:** This first-in-human study demonstrates that tumor-associated hK2 can be identified and targeted using h11B6 as a platform as the h11B6 antibody selectively accumulated in mCRPC metastases with mass-independent clearance kinetics. These data support the feasibility of hK2 as a target for imaging and hK2-directed agents as potential therapies in patients with mCRPC.

## Visual Abstract

**Figure:**
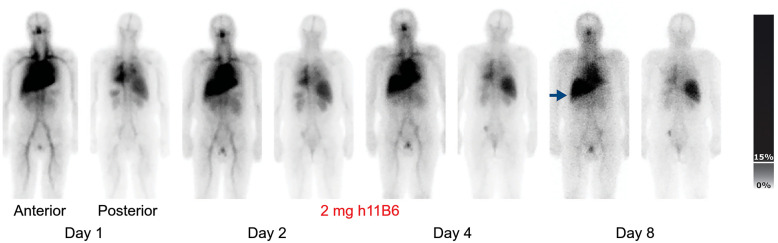

Patients with metastatic castration-resistant prostate cancer (mCRPC) have limited treatment options as novel androgen receptor (AR) inhibitors and cytotoxic therapies move to the castration-sensitive setting (1*,*^2^). Recently, the mCRPC treatment landscape has expanded with the approval of the targeted radiopharmaceutical therapy ^177^Lu-vipivotide tetraxetan (Pluvicto; Novartis), a systemic treatment that delivers radiation directly to the tumor cell, providing a novel mechanism for mCRPC treatment (3). Administration of such therapies requires a well-defined tumor-specific target to support effective payload delivery and minimal toxicity. In addition, other tumor-directed therapies, such as immunotherapies with chimeric antigen receptor T cells or T-cell–sequestering antibodies and antibody–drug conjugates, are promising avenues for drug development for mCRPC (4*,*^5^). Prostate-specific membrane antigen (PSMA) is the only cell surface target to be leveraged for clinical benefit in men with advanced prostate cancer; however, PSMA expression is heterogeneous among patients and even within patients on a lesion-to-lesion basis (6). PSMA is also putatively expressed in normal tissues such as the salivary and lacrimal glands, which can lead to off-tumor but on-target side effects in nonprostate tissue (6). Finally, patients who progress through PSMA-directed therapy may develop disease that does not express PSMA. New targets are therefore needed to address these shortcomings and to advance the treatment options for a disease as biologically dynamic and diverse as mCRPC.

Human kallikrein 2 (hK2; HUGO Gene Nomenclature Committee–approved gene symbol *KLK2*) is a trypsinlike antigen produced by columnar prostate epithelial cells with expression driven by AR signaling in a manner identical to that of the closely related gene encoding prostate-specific antigen (HUGO Gene Nomenclature Committee–approved gene symbol *KLK3*) (7–10). hK2 is both secreted and membrane-bound; however, unlike prostate-specific antigen, circulating hK2 is found at exceptionally low levels, where it can be bound by multiple protease inhibitor complexes (7*,*^8^*,*^11^). It has been shown that higher hK2 expression correlates with increased cell proliferation and lower apoptosis in castration-resistant prostate cancer specimens, thereby modulating the growth of castration-resistant disease (12). Additionally, a study reported 80% of mCRPC bone lesions to be AR-driven based on high expression of AR gene signatures, including both *KLK2* and *KLK3* (13). Increased AR signaling activity has demonstrated increased hK2 expression but decreased PSMA expression in multiple prostate cancer models (8*,*^14^*,*^15^). Specifically, treatment with the α-particle emitter ^225^Ac generated a feed-forward mechanism with increased AR signaling and hK2 expression (8). This could impact therapeutic outcomes of targeting hK2 versus PSMA in mCRPC, because reactivation of AR signaling is commonly associated with mCRPC, despite castration serum levels of androgen. Indeed, preclinical observations in animal models showed improved survival after a single administration of hK2-targeting ^225^Ac, suggesting a therapeutic benefit of targeting hK2 (8). Finally, hK2 expression shows prostate specificity and is relatively homogeneously expressed across disease stages, from localized to metastatic prostate cancer (16). Together, these data suggest hK2 to be a promising target for treatment.

In preclinical studies, reduction in tumor volume along with prolongation of survival were observed in mouse models of prostate cancer treated with the ^225^Ac-radiolabeled humanized anti-hK2 monoclonal antibody, h11B6, where h11B6 bound only membrane-associated hK2 (8*,*^17^). This phase 0 first-in-human study evaluated the biodistribution, normal-tissue dosimetry, and tumor targeting of ^111^In-radiolabeled h11B6 ([^111^In]-DOTA-h11B6) to evaluate hK2 as a target in patients with mCRPC.

## MATERIALS AND METHODS

### Study Design and Participants

This was a phase 0 imaging study conducted to evaluate the biodistribution, normal-tissue dosimetry, and tumor targeting of [^111^In]-DOTA-h11B6 (ClinicalTrials.gov identifier NCT04116164). After written informed consent for study participation was provided by the patients, screening of eligible participants was conducted 14 d before administration of the study agent. Participants at least 18 y of age with progressive measurable/evaluable mCRPC as defined by Prostate Cancer Working Group 3 criteria were prospectively enrolled in the study. Eligible participants had an Eastern Cooperative Oncology Group performance status of 1 or less, a Karnofsky performance scale score of 70 or greater, castrate testosterone levels less than 50 ng/dL, and metastatic disease documented by CT, PET/CT, MRI, or a radionuclide (^99m^Tc-methylene diphosphonate or ^18^F-NaF) bone scan. Participants with lesions identified by PSMA PET only and participants with pure small-cell or neuroendocrine prostate cancer were excluded. Participants could not have any condition that could impair the ability to comply with study procedures and could not have received radiotherapy or immunotherapy within 30 d, a therapeutic radioactive isotope within 28 d, or a single fraction of palliative radiotherapy within 14 d of administration of the study agent. Participants with a known allergy to antibodies were also excluded. The study was performed under a U.S. Food and Drug Administration–reviewed exploratory investigational new drug application and was approved by the Memorial Sloan Kettering Cancer Center Investigational Review Board, and all participants signed an informed consent form. Study data were collected and processed with adequate precautions to ensure confidentiality and compliance with data privacy protection laws and regulations.

The study enrolled participants in cohort 1, with the option to expand into an additional cohort at the discretion of the sponsor on the basis of findings from cohort 1. The focus of cohort 1 was to identify the optimal mass of [^111^In]-DOTA-h11B6, which was defined as the mass at which there is minimal accumulation of radioactivity in parenchymal organs (liver, lung, spleen) and maximal accumulation in mCRPC lesions as assessed by imaging. Data collected from cohort 1 were used to estimate the serum clearance, the normal-tissue dosimetry, and the most favorable mass amount of [^111^In]-DOTA-h11B6. Participants in cohort 1 received a single administration of [^111^In]-DOTA-h11B6 (185 MBq) as a slow intravenous bolus over 1 min ± 30 s followed by a saline flush through an indwelling peripheral intravenous catheter.

Participants in cohort 1 were divided into 3 subcohorts (1A, 1B, and 1C). Participants in subcohort 1A received 2 mg of antibody ([^111^In]-DOTA-h11B6 only) followed by imaging and biodistribution studies. Participants in subcohort 1B received 10 mg of antibody (8 mg of h11B6 and 2 mg of [^111^In]-DOTA-h11B6) with subsequent imaging and biodistribution studies. Subcohort 1C was to receive 20 mg of antibody (18 mg of h11B6 and 2 mg of [^111^In]-DOTA-h11B6) followed by imaging.

The primary endpoints of the study were safety and [^111^In]-DOTA-h11B6 dosimetry, including serum clearance, optimal mass, and [^111^In]-DOTA-h11B6 biodistribution. Secondary and exploratory endpoints included pharmacokinetic parameters and serum hK2 levels, respectively.

### [^111^In]-DOTA-h11B6 Imaging Agent

The macrocyclic chelator DOTA was conjugated to h11B6 (DiaProst AB) through incubation in a p-SCN-Bn-DOTA solution (MacroCyclics Inc.) at 25°C for 20 h. [^111^In]-DOTA-h11B6 was prepared by incubating ^111^In in acetate buffer for 2 h at 37°C with DOTA-h11B6. The reaction mixture was purified and tested for sterility, endotoxins, and radiochemical purity. Immunoreactivity was measured through a validated immunoassay. Preclinical testing of [^111^In]-DOTA-h11B6 demonstrated radiochemical purity of more than 95% and immunoreactivity consistently greater than 80%. In vitro studies confirmed antigen-mediated antibody internalization as previously reported (8).

### Assessments

Anterior and posterior planar ^111^In whole-body images were obtained immediately after study agent administration (0–4 h) and on day 2 (24 ± 6 h), days 3–4 (42–78 h), and days 6–9 (114–198 h). A SPECT/CT scan from the inferior portion of the lungs to the kidneys was performed at one or more of these time points. For all time points except 0–4 h, participants were instructed to void urine before imaging. Imaging was performed on a dual-head hybrid γ-camera (Siemens Intevo SPECT/CT; Siemens Healthcare).

Blood samples were collected before treatment; 15, 30, 60, 120, and 240 min (±5 min) after study agent administration; at each imaging session; and at the 2-wk follow-up visit to measure the radioactivity and/or serum concentrations of h11B6. Vital signs (blood pressure, pulse, respiratory rate, temperature, and weight) were obtained before and up to 2 h after study agent administration as well as at imaging. Complete blood count and comprehensive metabolic panels were obtained at baseline and at follow-up.

Serum samples were measured in duplicate using an NaI (Tl) γ-well–type detector (Wallac Wizard 1480 automatic γ-counter; PerkinElmer) together with appropriate standards to assess radioactivity. The measured radioactivity concentrations were converted to a percentage of injected dose per liter, and serum volume of distribution was estimated as 100% per time-zero intercept of the percentage of injected dose per liter. Serum concentrations of h11B6 were determined using a validated immunoassay.

Absorbed radiation doses to normal tissues from [^111^In]-DOTA-h11B6 were estimated on the basis of serum clearance of radioactivity and by image analysis of tissue uptake and retention of radioactivity over time. Data of the time-integrated activity coefficient derived from this analysis were used with the U.S. Food and Drug Administration–approved software package application OLINDA/EXM version 2.0 (Hermes Medical Solutions) to estimate absorbed radiation doses to a panel of normal tissues using the MIRD method.

Accumulation of radioactivity was defined as an increasing ratio of tumor-to-nontumor signal over time in known sites of disease. The number of lesions that demonstrated accumulation of radioactivity was also measured.

Adverse events were assessed using the National Cancer Institute Common Terminology Criteria for Adverse Events version 5.0, and participants were monitored for side effects during infusion and at every follow-up visit for up to 2 wk after agent administration. Participants were contacted 1–2 d after study agent administration to ascertain occurrence of any treatment-emergent adverse events when symptoms were not assessed during an on-site visit.

### Statistical Analysis

Descriptive statistics were calculated for key parameters, reported as the mean value and SD or SE.

## RESULTS

### Participant Population

Of the 22 participants included in this analysis, characteristics were comparable across the 2 subcohorts (16 in subcohort 1A and 6 in subcohort 1B; Table 1; Supplemental Table 1; supplemental materials are available at http://jnm.snmjournals.org). Since the optimum mass amount was determined in the first 2 subcohorts, the study did not accrue to subcohort 1C. SPECT/CT imaging identified tumors located in bone (*n* = 14), liver (*n* = 3), and lymph nodes (*n* = 3) (Table 1). ^111^In-administered activities were similar between cohorts with a mean ± SD of 205 ± 16 MBq and 201 ± 7 MBq in subcohorts 1A and 1B, respectively.

**TABLE 1.** Participant Characteristics Across Subcohorts

Subcohort	Patient	Age (y)	Weight (kg)	ECOG PS	Gleason score	Baseline PSA (ng/mL)	Primary metastatic tumor location*	Targeting location	Administered activity (MBq)
1A	001	84	92	0	9 (5 + 4)	19.7	Bone	Yes	218
	002	68	92	0	8 (3 + 5)	22.6	Liver	Yes	221
	003	70	85	0	7 (3 + 4)	40.5	Bone	Yes	202
	007	80	80	0	7 (4 + 3)	13.3	Bone	Yes	206
	009	70	111	0	6 (3 + 3)	43.5	Bone	Yes	213
	013	69	85	0	9 (5 + 4)	5.34	Nodes	Yes	210
	014	75	86	1		2.78	Bone	Yes	216
	016	75	60	1	9 (4 + 5)	1.34	Bone	Yes	199
	017	66	103	1	7 (3 + 4)	113	Bone	Yes	199
	018	61	100	1	10 (5 + 5)	73.8	Bone	Yes	196
	019	66	104	0	7 (3 + 4)	84.4	Bone	Yes	202
	020	72	90	0	9 (4 + 5)	2.44	Bone	Yes	203
	022	60	158	1	9 (4 + 5)	39.4	N/A^†^	N/A^†^	155
	023	81	92	0	7 (3 + 4)	10.5	Nodes	Yes	221
	025	62	111	0	7 (4 + 3)	9.38	Bone	Yes	197
	027	75	84	1	9 (4 + 5)	66.6	Liver	No	218
1B	004	68	85	0	9 (4 + 5)	4.96	Bone	Yes	206
	005	79	79	0	6 (3 + 3)	49.4	Bone	Yes	196
	006	82	56	1	7 (4 + 3)	124	Nodes	Yes	193
	008	56	91	0	9 (4 + 5)	507	Bone	Yes	201
	011	65	89	0	9 (4 + 5)	8.99	Liver	No	195
	015	72	109	0	9 (4 + 5)	1.33	None^‡^	None	213

* Tumor location is based on conventional imaging or PSMA PET.

† Participant was unable to complete remaining imaging per protocol because of noncompliance and discontinued study early. No results on tumor location or targeting are available.

‡ Participant had no obvious disease on imaging.

ECOG PS = Eastern Cooperative Oncology Group performance status; PSA = prostate-specific antigen; N/A = not available.

Subcohort 1A received 2 mg of protein, and subcohort 1B received 10 mg of protein.

### Safety

All participants who received the study agent were included in the safety and tolerability analysis. [^111^In]-DOTA-h11B6 was well tolerated. Of the 22 participants enrolled, 2 participants (9.1%) in subcohort 1A experienced treatment-emergent adverse events, which were not treatment-related, including 1 case of grade 3 tumor-related bleeding and 1 case of grade 2 hypertension. The participant with tumor-related bleeding discontinued postinjection imaging. No infusion reactions or anaphylactoid reactions were reported.

### Biodistribution, Pharmacokinetics, and Radioactivity Clearance

**FIGURE 1.**
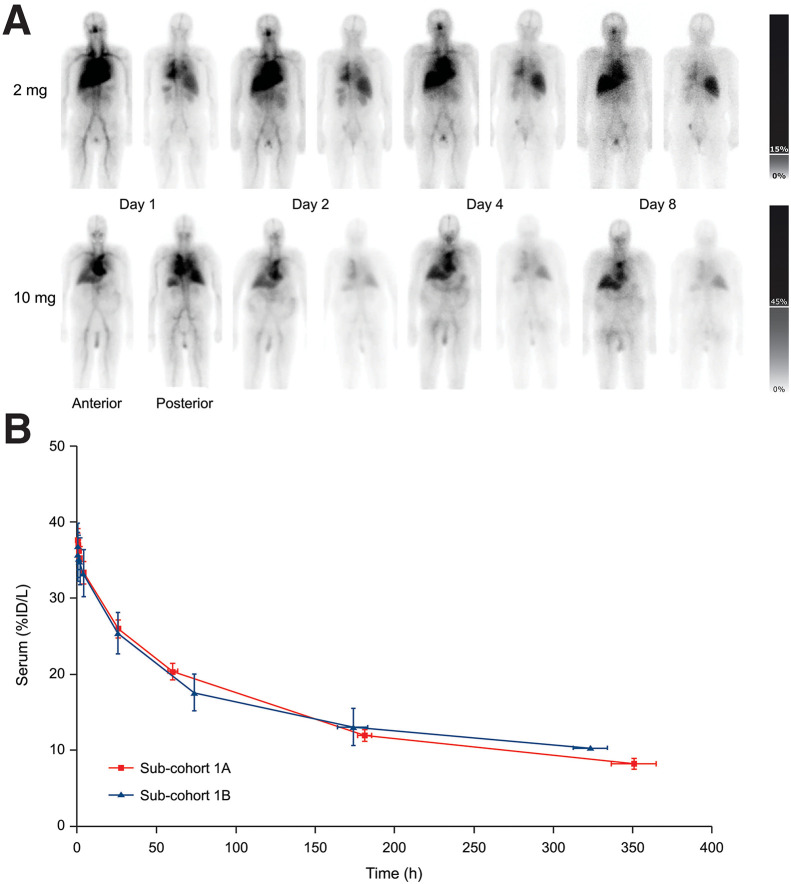
(A) Representative anterior and posterior whole-body images over time showing biodistribution after administration of 2 mg (top) or 10 mg (bottom) of [^111^In]-DOTA-h11B6. Bars on right represent percentage of maximum pixel value over all images. (B) Average serum clearance kinetics are based on decay-corrected percentage of injected ^111^In activity (percentage of injected dose per liter ± SE) across subcohort 1A (2 mg, *n* = 15) and subcohort 1B (10 mg, *n* = 6). %ID/L = percentage of injected dose per liter.

Serial whole-body images showed gradual clearance of radioactivity from the vascular compartment and gradual though normal accumulation in the liver at later time points. There was no evidence of increased physiologic [^111^In]-DOTA-h11B6 accumulation in bones, salivary glands, or the thyroid at either antibody mass level. Time-integrated activity analyses confirmed predominant localization of [^111^In]-DOTA-h11B6 in the vascular compartment rather than in critical normal organs (Table 2), and absorbed radiation doses of critical normal organs were low for both cohorts (Table 3). A complete list of absorbed radiation doses of normal organs is included in Supplemental Table 2. A relationship between administered antibody mass and biodistribution was not observed (Fig. 1A). Serum clearance kinetics showed a gradual decline in radioactivity over time (Fig. 1B), with no apparent difference between the 2-mg antibody mass (subcohort 1A) and 10-mg antibody mass (subcohort 1B), suggesting independence of antibody mass. The mean volume of [^111^In]-DOTA-h11B6 distribution ± SE was 2.82 ± 0.13 L and 2.87 ± 0.26 L for subcohort 1A (*n* = 15) and subcohort 1B (*n* = 6), respectively.

**TABLE 2.** Time-Integrated Activity Coefficients for Key Organs by Subcohort A (*n* = 15*) and Subcohort 1B (*n* = 6)

Subcohort	Time (h)		
	Kidney	Liver	Red marrow
1A	0.99 (0.05)	13.0 (0.6)	5.03 (0.19)
1B	0.87 (0.10)	12.8 (1.5)	4.69 (0.54)

* One participant was unable to complete remaining imaging per protocol because of noncompliance and discontinued study early.

Data are mean with SE in parentheses. Subcohort 1A received 2 mg of protein, and subcohort 1B received 10 mg of protein.

**TABLE 3.** Absorbed Radiation Doses for Key Organs by Subcohort 1A (*n* = 15*) and Subcohort 1B (*n* = 6)

Subcohort	Absorbed dose/activity (mGy/MBq)			
	Kidney	Liver	Red marrow	Total body
1A	0.23 (0.01)	0.45 (0.02)	0.17 (0.01)	0.12 (0.003)
1B	0.22 (0.02)	0.46 (0.05)	0.17 (0.01)	0.12 (0.01)

* One participant was unable to complete remaining imaging per protocol because of noncompliance and discontinued study early.

Data are mean with SE in parentheses. Subcohort 1A received 2 mg of protein, and subcohort 1B received 10 mg of protein.

**FIGURE 2.**
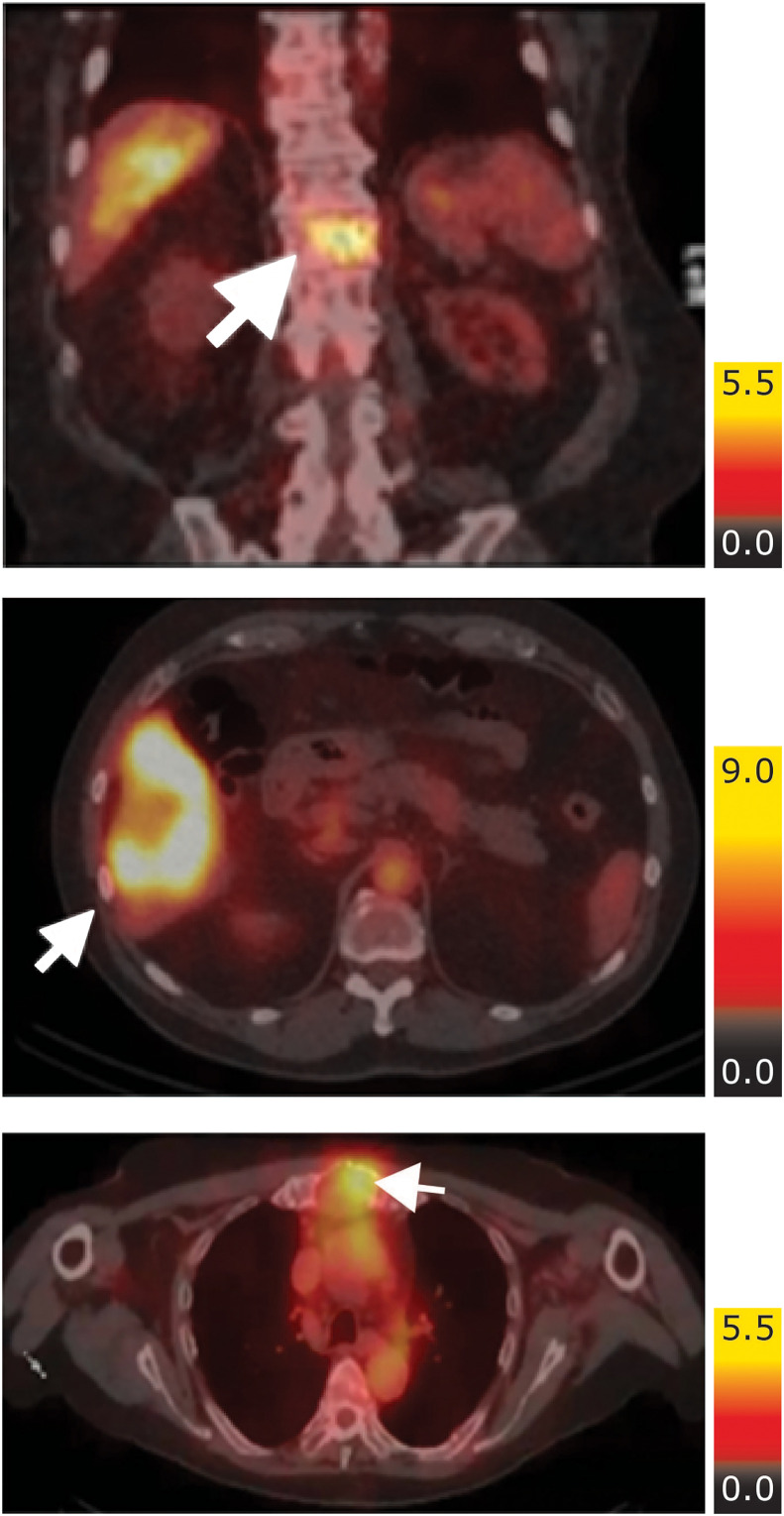
Representative images showing tumor uptake of [^111^In]-DOTA-h11B6 administered at 2 mg (top 2 images) or 10 mg (lower image). Arrows denote lesions.

Targeting of [^111^In]-DOTA-h11B6 to known lesions was observed in 18 participants via SPECT/CT (Fig. 2) and was confirmed via ^68^Ga-PSMA-11 PET/CT (data not presented). In the 4 participants with no evidence of lesion targeting, 1 participant had no evidence of disease by PSMA PET and 1 had incomplete imaging.

## DISCUSSION

This first-in-human study demonstrated the favorable distribution of [^111^In]-DOTA-h11B6 in participants with mCRPC and highlighted the feasibility of hK2-directed imaging. Tumor uptake was noted in 18 of 22 participants. Of the 4 participants with no evidence of tumor targeting, 1 had no evidence of disease by PSMA PET and 1 participant had incomplete imaging. Tumor accumulation of [^111^In]-DOTA-h11B6 was visually observed approximately 4 d after agent administration and was more evident via imaging with increasing time after injection.

As with other monoclonal antibodies, serum clearance kinetics and time–activity analyses demonstrated antibody localization predominantly to the vascular compartment, with clearance characteristics comparable to those of other intact humanized IgGs (18*,*^19^). Imaging data further supported vascular compartment localization, with no evidence of significant parenchymal targeting or any extratumoral antigen sink. Importantly, the antibody mass amounts studied (2 and 10 mg) had no effect on serum clearance, volume of distribution, or tumor targeting.

[^111^In]-DOTA-h11B6 was tolerated in participants with mCRPC. Two treatment-emergent adverse events were observed; however, these events were not related to the study treatment. The patient who experienced tumor-related bleeding discontinued the study early. Normal-organ and whole-body absorbed radiation doses were well within the limits of use for imaging radiopharmaceuticals. Unlike PSMA-targeted agents that are accompanied by an uptake in the lacrimal and salivary glands and in the renal tubules, which can lead to dose-limiting toxicity (20), targeting of hK2 with [^111^In]-DOTA-h11B6 showed no significant uptake in the salivary glands and a modest renal uptake (0.22–0.23 mGy/MBq); therefore, low radiation doses to these organs is expected. Phase 1 studies assessing hK2-targeting therapies, including ^225^Ac radioimmunotherapy, are currently ongoing and may further support hK2 as a therapeutic target in advanced prostate cancer (21). As off-target localization to parenchymal organs and bone marrow is a potential area of concern, especially for radioactive payloads, hK2-targeting radiolabeled agents will need careful monitoring. To this end, the risks of myelosuppression, renal and liver toxicity, and gastrointestinal effects have been identified with appropriate mitigation strategies in the ongoing phase 1 study assessing JNJ-6240 ([^225^Ac]-DOTA-h11B6) (21).

The tumor-targeting specificity of [^111^In]-DOTA-h11B6, its mass-independent biodistribution, and its tolerable safety profile together suggest a role for h11B6 as a carrier for targeted delivery of a conjugated payload for the treatment of mCRPC. Through characterization of h11B6 biodistribution in human participants, this study further adds to previous preclinical prostate cancer studies (8*,*^22^) by validating the expression of hK2 in mCRPC, providing data to support the use of h11B6 in a clinical setting.

## CONCLUSION

This phase 0 trial demonstrated that [^111^In]-DOTA-h11B6 was well tolerated with mass-independent biodistribution and tumor targeting in mCRPC. These data not only confirm the validity of hK2 as both an imaging and a potential therapeutic target for mCRPC but also support the continued investigation of h11B6 for directed delivery of payloads such as therapeutic radionuclides to address an unmet need for additional mCRPC treatment options.

## DISCLOSURE

This work was supported by Janssen Research & Development, LLC, and SpectronRX, Inc. Neeta Pandit-Taskar reports honoraria from Actinium Pharmaceuticals; a consulting or advisory role for Illumina, Progenics, Telix, and Lantheus; a Speakers Bureau association for Actinium Pharmaceuticals and Telix; research funding (institutional) from Bayer Health, Bristol Myers Squibb, Clarity Pharmaceuticals, Imaginab, Janssen Research & Development, LLC, Regeneron, Ymabs, and Innervate; and travel support from AstraZeneca and Bayer. Joseph O’Donoghue reports consulting for Janssen Research & Development, LLC; Invicro, LLC; and Curadh MTR. Dushen Chetty reports stock and other ownership interests for Novartis AG and is an employee of Janssen Research & Development, LLC. Steven Max, Margaret Yu, and Michael Russell report stock and other ownership interests for, and are employees of, Janssen Research & Development, LLC. Danielle Wanik reports research funding from Janssen Research & Development, LLC (institutional), and is an employee of Invicro LLC. Ohad Ilovich is an employee of Invicro LLC. Chaitanya Divgi reports a consulting or advisory role for Janssen Research & Development, LLC, and SpectronRX, Inc. Michael Morris reports stock and other ownership interests for Doximity; a consulting or advisory role for Lantheus Medical Imaging, AstraZeneca, Amgen, Daiichi, Convergent Therapeutics, Pfizer, ITM Isotope Technologies Munich, Clarity Pharmaceuticals, Blue Earth Diagnostics, POINT Biopharma, Telix Pharmaceuticals, Progenics, and Z-α; research funding from Bayer (institutional), Progenics (institutional), Corcept Therapeutics (institutional), Roche/Genentech (institutional), Janssen Research & Development, LLC (institutional), Celgene (institutional), Novartis (institutional), and Astellas Pharma (institutional); and travel, accommodations, and expenses from AstraZeneca, APCCC, and Memorial Sloan Kettering Cancer Center. No other potential conflict of interest relevant to this article was reported.

## Notes

KEY POINTS **QUESTION:** Can hK2 expression be leveraged as a prostate cancer imaging and therapeutic target in humans? **PERTINENT FINDINGS:** This first-in-human study demonstrated that the [^111^In]-radiolabeled anti-hK2 antibody, [^111^In]-DOTA-h11B6, selectively targeted mCRPC tumors without safety concerns. **IMPLICATIONS FOR PATIENT CARE:** This study highlights the feasibility of hK2 as a prostate cancer target and supports continued investigation of h11B6 or other hK2-targeting agents in the treatment of mCRPC.
